# Prosthetic Complications of Single Screw-Retained Implant-Supported Metal–Ceramic Fixed Prostheses: A Retrospective Observational Study

**DOI:** 10.1155/2024/9242928

**Published:** 2024-08-13

**Authors:** Cristina Palma-Carrió, Andrea Macconi, Andrea Rubert-Aparici, Paula Vidal-Peiró, Isabel Menéndez-Nieto, Juan Antonio Blaya-Tárraga

**Affiliations:** ^1^ Faculty of Dentistry European University of Valencia, Valencia, Spain; ^2^ European University of Valencia, Valencia, Spain; ^3^ Clinical Department Faculty of Dentistry European University of Valencia, Valencia, Spain

## Abstract

**Purpose:**

To analyze prosthetic complications of single screw-retained implant-supported metal–ceramic fixed prostheses (SSIMCFPs).

**Materials and Methods:**

A total of 457 medical records of patients treated with implants at the University Dental Clinic of the European University of Valencia from 2016 to 2022 were reviewed. Of the 335 SSIMCFPs evaluated, 222 were included. The following data were collected from medical records: age, sex, prosthesis location, implant diameter, type of antagonist, date of prosthesis placement, type of prosthetic complications, and the date of the occurrence of complications. Statistical analysis was estimated at the patient level with a simple binary logistic regression and at the prosthesis level, a simple logistic regression with generalized estimating equation models (*p*  < 0.05).

**Results:**

A total of 222 SSIMCFPs were placed in 159 patients. The prevalence of complications was 23.3% at the patient level, equivalent to 21.6% of SSIMCFPs. A total of 48 complications were collected; screw loosening was the most frequent complication (16.2%), followed by ceramic fracture (3.1%), screw fracture (1.8%), and implant fracture (0.5%). There were no cases of abutment fracture. The mean time of the loosening of the screw was 10.5 months and ceramic fractures at 6.9 months. The factors that most influenced the occurrence of prosthetic complications were posterior position (*p*  < 0.001), implant diameter from 3.5 to 4.8 mm (*p*  < 0.01), and lower arch position (*p*  < 0.05).

**Conclusions:**

The most frequent complication of SSIMCFP was loosening of the screw followed by ceramic fracture. The appearance of these complications usually occurred during the first year after SSIMCFP placement. Factors related to the occurrence of complications were mandibular posterior location and implant diameter from 3.5 to 4.8 mm.

## 1. Introduction

Single crowns on implants have become the treatment of choice for replacing missing teeth [1]. However, these treatments are not free from complications. Technical complications represent those related to laboratory-fabricated parts, such as fracture and loosening of the veneering materials, while mechanical complications represent complications related to prefabricated parts, such as implant fracture or failure of abutments and screws [2]. Prosthetic complications (technical and mechanical) do not necessarily lead to prosthetic failure but may result in a higher number of appointments and repairs following prosthesis placement [3].

Prosthesis complications may occur during the clinical life of the implant; however, in the literature, there are few articles [4, 5, 6, 7, 8, 9, 10] that study the prosthetic complications of implant-supported restorations, and very few studies [11, 12, 13] focus on the complications of single crowns. The most frequent complication is screw loosening, and although this is not a serious complication, if it occurs repeatedly, it can affect the success of the treatment and patient satisfaction [13]. In a systematic review, Jung et al. [14] reported that technical complications reached a cumulative incidence of 8.8% for screw loosening, 4.1% for loss of retention in cemented crowns, and 3.5% for fracture of the material, with no statistically significant differences between screw-retained and screw-cemented crowns.

The hypothesis of this study was SSIMCFP complications are related with risk factors. The primary objective of this retrospective study is to determine the prevalence of prosthetic complications in patients treated with SSIMCFP. The secondary objective is to determine in what period of time these complications appear and tertiary to relate the presence of prosthetic complications to factors associated (age, sex, implant diameter, arch, position, and antagonist).

## 2. Material and Methods

This retrospective observational study was conducted following the criteria of Strengthening the Reporting of Observational studies in Epidemiology (STROBE) [15]. Participants gave informed consent and permission for data release before participating in the study. The study was conducted following the deontological standards recognized by the Declaration of Helsinki and following the recommendations of Good Clinical Practice of the European Community. The research protocol was submitted to the Research Ethics Committee of the European University, which issued a favorable report on 26/01/2023 with the code CIPI/23.003.

### 2.1. Sample Selection

Sample selection was carried out by two reviewers (C.P.C. and A.M.) at the University Dental Clinic of the European University of Valencia. Through the computers of the university clinic, 457 medical records of patients treated with implants from 2016 to 2022 were reviewed.

### 2.2. Eligibility Criteria

The inclusion criteria were as follows: (1) patients with at least one SSIMCFP; (2) patients treated at the University Dental Clinic of the European University of Valencia from 2016 to 2022; and (3) patients older than 18 years with periodontal follow-up.

The exclusion criteria were as follows: (1) patients with incomplete medical history; (2) patients without prosthetic rehabilitation or with a follow-up period of less than 6 months; and (3) patients with a lack of radiological follow-up.

### 2.3. Data Collection

Data collected from the patients' medical records were as follows:

- Patient-related data: medical history number, age, and sex (male/female)
- SSIMCFP and implant-related data: arch (maxillary/mandibular), prosthesis location (anterior/posterior), implant diameter, and type of antagonist (natural dentition, denture or implant-supported prosthesis, overdenture, removable prosthesis, and absence of antagonist)
- Date of prosthesis placement
- Prosthetic complications related to SSIMCFP [9]: screw loosening, screw fracture, abutment fracture, implant fracture, and ceramic fracture
- Date of the occurrence of complications

The types of abutment and screws, materials used in the prostheses, and bruxism were excluded from our data collection because such information is sometimes missing from patients' medical records.

### 2.4. Statistical Analysis

Statistical analysis was performed using SPSS software (version 25, SPSS Inc., Chicago, IL, USA). At the patient level, a simple binary logistic regression was estimated for each dependent variable (probability of total complications, screw loosening, and ceramic fractures) as a function of independent factors (sex and age). The model estimates the coefficients and the unadjusted odds ratio (OR), together with the 95% confidence interval. At the SSIMCFP level, a *simple logistic regression with* generalized estimating equation models was estimated for each dependent variable (probability of total complications, screw loosening, and ceramic fractures) as a function of SSIMCFP characteristics. The model estimates the coefficients and the crude odds ratio (OR), together with the 95% confidence interval through the Wald chi-squared statistic. This method is justified by the intrasubject correlation (several SSIMCFPs come from the same patient). Based on the significant variables (*p* < 0.05) and relevant variables (*p* < 0.1), a multiple model was estimated to obtain adjusted ORs.

## 3. Results

A total of 335 SSIMCFPs were evaluated, of which 36 were discarded because the clinical history was not complete, 57 for lack of follow-up, 12 for follow-up of less than 6 months after prosthesis placement, and eight for lack of radiographic follow-up. A total of 222 SSIMCFPs were included in the study.

### 3.1. Descriptive Results

The study sample comprised 159 patients, 81 (50.9%) women and 78 (49.1%) men with a mean age of 53.8 ± 13.2 years and a range of 20–78 years. Of the 222 SSIMCFPs studied, 115 (51.8%) were placed in the maxilla and 107 (48.2%) in the mandible, with a total of 23 (10.4%) SSIMCFPs in the anterior sector and 199 (89.6%) in the posterior sector. The implants were classified according to their diameter in three categories: (1) diameter from 3 to 3.4 mm (47 implants, 21.2%), (2) diameter from 3.5 to 4.8 mm (144 implants, 64.9%), and (3) diameter equal to 5 mm or greater than 5 mm (31 implants, 14.0%).

**Figure 1:**
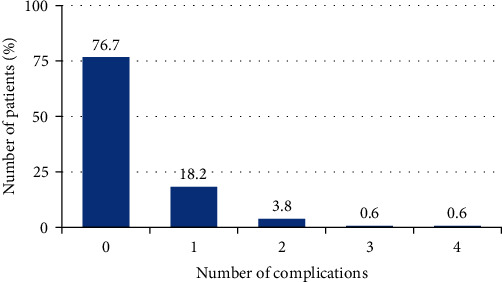
Number of complications per patient.

At the patient level, 76.7% (122 of 159 patients) did not experience any prosthetic complications. On the other hand, 18.2% (29 patients) experienced a single complication. In addition, 3.8% (six patients) were recorded as experiencing two complications, while one patient (0.6%) had three complications, and another patient (0.6%) had four complications (Figure 1).

**Figure 2:**
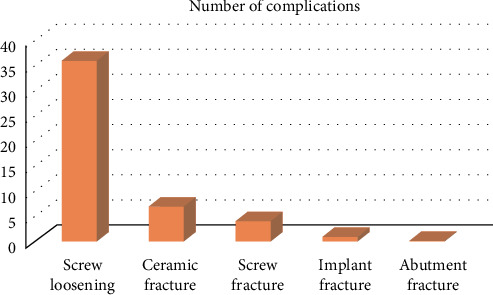
Number of complications.

At the prosthesis level, of the 222 SSIMCFPs included in the study, 48 (21.6%) presented complications, while 174 (82.4%) did not experience any prosthetic complications. For the distribution of these complications, it was observed that 16.1% corresponded to screw loosening, 3.1% presented ceramic fracture, 1.8% experienced screw fracture, 0.5% suffered implant fracture, and none suffered abutment fracture (Figure 2).

The mean time of the loosening of the screw was 10.5 months and ceramic fractures at 6.9 months, screw fracture at 23.4 months, and implant fracture at 44 months. Regarding screw loosening, secondary complications were observed in five of the 31 SSIMCFPs. Once the screw was retightened, it took 14 months for the second loosening to occur.

### 3.2. Statistical Results

Regarding the factors that influence the appearance of complications at the patient level, neither sex nor age presented a significant association (*p* > 0.01) (Table 1).

The factors that most influenced the occurrence of prosthetic complications were posterior position (*p* < 0.001), implant diameter from 3.5 to 4.8 mm (*p* < 0.01), and lower arch position (*p* < 0.05) (Table 2).

Factors associated were investigated in the most prevalent complications: screw loosening and ceramic fracture. The factors that had a statistically significant influence on screw loosening were posterior location (*p* < 0.001) and implant diameter from 3.5 to 4.8 mm (*p* < 0.001) (Table 3). Regarding ceramic fracture, the most important factor was the location in the posterior region (*p* < 0.01) (Table 4).

## 4. Discussion

Few articles have evaluated the prosthetic complications of SSIMCFPs. The results of this study indicate that the prevalence of complications related to SSIMCFPs is 21.6%. It is difficult to compare these results in terms of prevalence because most studies speak of complications in general, without considering the type of prosthesis. For example, Kourtis et al.[10], in an extensive retrospective clinical study with a follow-up time of up to 12 years, found an average prevalence of prosthetic complications of 9.52%. In this study, no distinction was made between the different implant-borne prostheses. In a systematic review carried out by Jung et al. [14], the incidence of technical complications in implant-supported single crowns was 8.8%, lower than what was found in this study.

In the present investigation, the main complications were loosening of the screw (16.2%) and fracture of the ceramic (3.1%), with the respective means occurring at 10.5 and 6.9 months after prosthesis placement. This is in accordance with the study by Lee et al. [5], where most complications occurred within 6 months of loading, accounting for 50.4% of the cases. Furthermore, this result agrees with the study of Wang et al. [16], which found that ceramic fracture occurred within the first 4–8 months. The recurrence of the same complication, screw loosening, occurred for the second time at 14 months after screw tightening. Again, this is in line with the results of Lee et al. [5], where the same complication occurred for the second time at 12 months. Other complications, such as fractures of the screw, implant, and abutment, tend to occur later, although a larger number of cases would be needed for the results to be statistically significant.

In this study, the risk factors statistically significantly associated with the occurrence of complications in SSIMCFPs were the posterior location (*p* < 0.001) of the SSIMCFP, followed by implant diameter from 3.5 to 4.8 mm (*p* < 0.01), and finally a lower arch location (*p* < 0.03).

Screw loosening was the most frequent complication, occurring in 16.2% of the cases, which is a result similar to that found by Wittneban et al. [4] (12.7%) and by De Boever et al. [17] (12%). The studies by Lee et al. [5] and Sailer et al. [9] reported a prevalence rate of screw loosening of 7.2% and 8.8%, respectively. However, studies such as Theoharidou et al. [18] and Goodacre et al. [7] state that screw loosening was also the main complication, but with an incidence of 3%, which is much lower compared to the present investigation.

The present study does not show a significant association between screw loosening and patient-related factors such as sex and age, coinciding with the results obtained by Lee et al. [5]. The variables that were statistically significantly related to screw loosening were the posterior position of the SSIMCFP (*p* < 0.001) and an implant diameter from 3.5 to 4.8 mm (*p* < 0.001). Again, both variables studied agree with the study of Lee et al. [5]. Similarly, in the study by Cho et al. [19], an increased risk in the molar region (12.3%) was observed with respect to the anterior region (7.7%). Wang et al. [16] states that anterior single tooth crowns experience half as much screw loosening as posterior crowns. However, Cho et al. [19] observed that screw loosening was more frequent in standard diameter implants than in wide diameter implants.

In the present investigation, the prevalence of ceramic fracture in SSIMCFPs was observed to be 3.1%, similar to what was found in other studies [9, 12]. Although these results suggest that ceramic fracture is not a very frequent complication, it is the most frequent prosthetic complication in studies such as Wittneben et al. [4] (20.31%), Brägger et al. [20] (10.4%), and Pjetursson et al. [8] (7.8%), which differs from the results in the present study.

In this study, the association between the posterior position of the SSIMCFP and the risk of ceramic fracture was statistically significant (*p* < 0.01). However, Wittneben et al. [4] did not find a significant relationship between implant position and ceramic fracture.

In relation to screw fracture, it was observed that it occurred in only 1.8% of the 222 SSIMCFPs analyzed, such as the 1.3% in the study by Pjetursson et al. [8]. On the other hand, Jung et al. [12] found the rates lower than 0.35% in their 2008 study.

In this study, implant fracture occurred in one case, with a prevalence of 0.5%. Similar data were reported by Pjetursson et al. [8], with a fracture rate of 0.5% at 5 years. Regarding risk factors, it is not possible to identify a statistically significant relationship. Some authors, such as Goiato et al. [21], have identified a higher incidence of fracture in implants with smaller diameters. Bruxism has also been identified by Tosun et al. [22] as a risk factor for implant fracture.

Finally, no cases of abutment fracture were reported (0%). This rate is consistent with previous studies by Jung et al. [12] and Nedir et al. [6], who reported low cumulative rates of 0.35% and 0.37%, respectively. However, studies such as Pjetursson et al. [8] and Sailer et al. [9] showed higher rates of 1.3% and 2%, respectively.

The main limitation of this study is its retrospective design. As a result, no information is given regarding the materials used in the prosthesis, types of abutment and screws, or the presence of bruxism. Therefore, long-term results should be sought in a prospective study.

## 5. Conclusions

1. The prevalence of complications at the patient level was 23.3%, equivalent to 21.6% of the SSIMCFPs. The most frequent complications were screw loosening (16.2%), followed by ceramic fracture (3.1%). Screw fracture (1.8%) and implant fracture (0.5%) were considered infrequent complications. There were no cases of abutment fracture.
2. The most frequent complications, screw loosening and ceramic fracture, usually manifest themselves within 6 months to 1 year, while less common complications tend to occur at later stages.
3. The occurrence of prosthetic complications shows a significant relationship with certain factors, such as posterior position (*p* < 0.001), use of implants with a diameter from 3.5 to 4.8 mm (*p* < 0.01), and mandibular location (*p* < 0.05). Specifically, screw loosening was more associated with posterior location (*p* < 0.001) and implant diameter from 3.5 to 4.8 mm (*p* < 0.001), while ceramic fracture occurred more frequently in the posterior sector (*p* < 0.01).

## Figures and Tables

**Table 1:** Association between the prevalence of total complications and independent factors: odds ratio (OR) estimation with simple binary logistic regression (Patient-related data).

Independent factors	OR	CI 95%	*p*
Sex			
Men	1	—	—
Women	0.67	0.32–1.40	0.286
Age			0.994
≤45a	1	—	—
46−55a	1.12	0.41–3.05	0.383
56−65a	1.05	0.36–3.11	0.925
>65a	1.14	0.40–3.28	0.804

**Table 2:** Association between the prevalence of total complications and independent factors: odds ratio (OR) estimation with simple binary logistic regression (SSIMCFP and implant-related data).

Factors	OR	95% CI	*p*
Arch			
Maxilla	1	—	—
Mandible	1.12	1.01–1.24	0.030 ^*∗*^
Sector			
Anterior	1	—	—
Posterior	1.22	1.15–1.29	<0.001 ^*∗∗∗*^
Diameter			0.009 ^*∗∗*^
3–3.5 mm	1	—	—
3.5–4.8 mm	1.16	1.05–1.27	0.003 ^*∗∗*^
≥5	1.14	0.98–1.33	0.099
Antagonist			0.861
Natural teeth	1	—	—
Implant-borne prosthesis	1.04	0.87–1.24	0.698
Fixed tooth-borne prosthesis	1.05	0.83–1.33	0.678

^*∗*^*p*  < 0.05;  ^*∗∗*^*p*  < 0.01;  ^*∗∗∗*^*p*  < 0.001.

**Table 3:** Association between loosening screw prevalence and independent factors: odds ratio (OR) estimation with simple binary logistic regression.

Factors	OR	95% CI	*p*
Arch			
Maxilla	1	—	—
Mandible	1.08	0.98–1.18	0.119
Sector			
Anterior	1	—	—
Posterior	1.17	1.11–1.23	<0.001 ^*∗∗∗*^
Diameter			<0.001 ^*∗∗∗*^
3–3.4 mm	1	—	—
3.5–4.8 mm	1.17	1.08–1.26	<0.001 ^*∗∗∗*^
≥5	1.15	1.01–1.32	0.040 ^*∗*^
Antagonist			0.603
Natural teeth	1	—	—
Implant-borne prosthesis	0.94	0.83–1.06	0.318
Fixed tooth-borne prosthesis	1.00	0.82–1.22	0.994

^*∗*^*p*  < 0.05;  ^*∗∗∗*^*p*  < 0.001.

**Table 4:** Association between the prevalence of ceramic fractures and independent factors: odds ratio (OR) estimation with simple binary logistic regression.

Factors	OR	95% CI	*p*
Arch			
Maxilla	1	—	—
Mandible	1.03	0.98–1.08	0.213
Sector			
Anterior	1	—	—
Posterior	1.04	1.01–1.06	0.007 ^*∗∗*^
Diameter			0.898
3–3.4 mm	1	—	—
3.5–4.8 mm	0.99	0.93–1.05	0.645
≥5 mm	0.99	0.91–1.08	0.810
Antagonist			0.238
Natural teeth	1	—	—
Implant-borne prosthesis	1.11	0.97–1.27	0.129
Fixed tooth-borne prosthesis	1.06	0.92–1.21	0.437

^*∗∗*^*p*  < 0.01.

## Data Availability

Data are available upon reasonable request.
